# In Utero Exposure to Alcohol and Tobacco and Electroencephalogram Power During Childhood

**DOI:** 10.1001/jamanetworkopen.2023.50528

**Published:** 2024-01-05

**Authors:** Nicolò Pini, Ayesha Sania, Shreya Rao, Lauren C. Shuffrey, J. David Nugent, Maristella Lucchini, Marco McSweeney, Christine Hockett, Santiago Morales, Lydia Yoder, Katherine Ziegler, Matthew S. Perzanowski, Nathan A. Fox, Amy J. Elliott, Michael M. Myers, William P. Fifer

**Affiliations:** 1 Department of Psychiatry, Columbia University Irving Medical Center, New York, New York; 2 Division of Developmental Neuroscience, New York State Psychiatric Institute, New York; 3 Department of Child and Adolescent Psychiatry, NYU Grossman School of Medicine, New York, New York; 4 Department of Human Development and Quantitative Methodology, University of Maryland, College Park; 5 Center for Pediatric & Community Research, Avera Research Institute, Sioux Falls, South Dakota; 6 Department of Pediatrics, University of South Dakota School of Medicine, Sioux Falls; 7 Department of Psychology, University of Southern California, Los Angeles; 8 Department of Environmental Health Sciences, Mailman School of Public Health at Columbia University, New York, New York; 9 Department of Pediatrics, Columbia University Irving Medical Center, New York, New York

## Abstract

**Importance:**

Prenatal alcohol exposure (PAE) and prenatal tobacco exposure (PTE) are risk factors associated with adverse neurobehavioral and cognitive outcomes.

**Objective:**

To quantify long-term associations of PAE and PTE with brain activity in early and middle childhood via electroencephalography (EEG).

**Design, Setting, and Participants:**

This cohort study included participants enrolled in the Safe Passage Study (August 2007 to January 2015), from which a subset of 649 participants were followed up in the Environmental Influences on Child Health Outcomes Program. From September 2018 through November 2022, EEG recordings were obtained at ages 4, 5, 7, 9, or 11 years. Data were analyzed from November 2022 to November 2023.

**Exposures:**

Maternal self-reported consumptions of alcohol and tobacco during pregnancy were captured at the recruitment interview and at up to 3 visits during pregnancy (20-24, 28-32, and ≥34 weeks’ gestation). Classifications of PAE (continuous drinking, quit-early drinking, and nondrinking) and PTE (continuous smoking, quit-early smoking, and nonsmoking) were previously obtained.

**Main Outcomes and Measures:**

EEG band powers (theta, alpha, beta, gamma) were extracted from the EEG recordings. Linear regression models were used to estimate the associations of PAE and PTE with EEG estimates.

**Results:**

The final sample included 649 participants (333 [51.3%] female) aged 4, 5, 7, 9, or 11 years. Children whose mothers were in the quit-early drinking cluster had increased alpha power (0.116 [95% CI, 0.023 to 0.209] μV^2^; *P* = .02) compared with individuals without PAE. The magnitude of this increase was approximately double for children exposed to continuous drinking (0.211 [95% CI, 0.005 to 0.417] μV^2^; *P* = .04). Children whose mothers were in the continuous smoking cluster had decreased beta power (−0.031 [95% CI, −0.059 to −0.003] μV^2^; *P* = .03) and gamma power (−0.020 [95% CI, −0.039 to −0.000] μV^2^; *P* = .04) compared with the nonsmoking cluster. In exploratory sex-stratified models, male participants in the quit-early PAE cluster had greater EEG power in the alpha band (0.159 [95% CI, 0.003 to 0.315] μV^2^; *P* = .04) compared with those with no PAE, and the difference was approximately double for male participants with continuous PAE (0.354 [95% CI, 0.041 to 0.667] μV^2^; *P* = .03). Male participants in the continuous PTE cluster had decreased beta (−0.048 [95% CI, −0.090 to − 0.007] μV^2^; *P* = .02) and gamma (−0.032 [95% CI, −0.061 − 0.002] μV^2^; *P* = .04) power compared with those with no PTE.

**Conclusions and Relevance:**

These findings suggest that even low levels of PAE and PTE were associated with long-term alterations of brain activity.

## Key Points

**Question:**

Are prenatal alcohol exposure (PAE) and prenatal tobacco exposure (PTE) associated with brain activity, measured via electroencephalography (EEG), in early and middle childhood?

**Findings:**

In this cohort study of 649 participants ages 4 to 11 years contributing 795 EEG recordings, PAE and PTE were associated with EEG power. PAE was associated with increased low-frequency brain activity, whereas PTE was associated with decreased high-frequency brain activity.

**Meaning:**

These findings support the public health message that any level of alcohol and/or tobacco consumption during pregnancy has a potentially harmful impact on brain development in the offspring.

## Teaser

This cohort study assesses long-term associations of prenatal alcohol exposure and prenatal tobacco exposure with brain activity in early and middle childhood via electroencephalography.

## Introduction

Many long-term associations of high levels of prenatal alcohol exposure (PAE) and prenatal tobacco exposure (PTE) with increased risk for adverse offspring neurobehavioral and cognitive outcomes have been established, including attention deficit hyperactivity disorder, decreased general cognitive functioning, and deficits in learning and memory.^1,2,3,4,5,6,7,8,9,10,11,12,13,14,15,16,17,18,19,20^ However, there is a paucity of studies investigating the roles of low or moderate levels of PAE and PTE, which are common in the general population.^4,5,21^ Understanding the long-term associations of quantity, timing, and combinations of PAE and PTE with brain function in early and middle childhood could shed light on mechanisms underlying the associations of such exposures with offspring adverse health, cognitive, and behavioral outcomes.^2,21^

During 2018 to 2020, 13.5% of pregnant people aged 18 to 49 years in the US reported current drinking, and 5.2% reported an episode of binge drinking in the past 30 days according to the Behavioral Risk Factor Surveillance System data collected by the Centers for Disease Control and Prevention.^22^ Approximately 1 in 14 pregnant people (7.2%) in the US in 2016 reported smoking tobacco during pregnancy. Smoking during pregnancy was most common among pregnant people aged 20 to 24 years.^23^ Despite this evidence, most published work investigating associations between in utero alcohol exposure and functional brain development focuses on heavy PAE and/or maternal chronic alcoholism^24,25,26,27,28,29,30^ or on cohorts including individuals diagnosed with fetal alcohol spectrum disorders.^31,32,33,34,35,36,37^ Most of these studies had limited sample sizes,^24,25,29,36,38^ or data collected at a single time point, ie, either in infancy^24,25,27,28,30,39^ or adolescence.^33,34,35,36,38,40^ Similar constraints apply to studies investigating PTE.^4,5,13,41,42,43,44^ Therefore, these findings are not easily translatable to the general population, given the lower but more widespread levels of PAE and PTE reported by pregnant individuals in the US.^22,23^

Research on PAE and PTE during pregnancy has demonstrated teratogenic effects of these agents, and even low exposures levels may have severe negative consequences for the development of the fetus and at the subsequent early stages of life.^45^ Our research teams have extensively investigated associations of adverse health outcomes and autonomic and central nervous systems vulnerability with PAE and PTE.^45,46,47,48,49^ Taken together, these findings highlight the susceptibility of the autonomic and central nervous systems to in utero exposure throughout the fetal and neonatal periods and the feasibility of using noninvasive markers of autonomic and central nervous systems functioning for detecting anomalies associated with PAE and PTE in multiple physiological control mechanisms.^50^ However, it is unclear whether functional alterations underlying these risk profiles are associated with PAE and PTE and whether they persist beyond the early stages of life.

This cohort study examines the long-term associations of patterns of PAE and PTE with brain activity quantified via EEG spectral analyses. EEG is one of the most exploited neuroimaging tools for conducting noninvasive investigations of the brain. Neural oscillations, often quantified via EEG power spectral density, are among the most common features. Such oscillations are observed at multiple spatial and temporal scales and have been shown to be prone to alterations elicited by a variety of experiences,^51^ although a limited number of studies have examined associations of PAE and PTE with EEG power. Hypersynchronous EEG is a convergent finding in infants with PAE.^24,25,27,52^ This evidence is thought to reflect alteration in γ-aminobutyric acid (GABA) receptor system modulation and glutamatergic system inhibition resulting in increased EEG power in the theta and alpha ranges.^53,54^ Conversely, PTE has been associated with alteration of the acetylcholine receptor subtypes and neurotransmitter system (eg, dopamine, norepinephrine, serotonin) involved in alertness and arousal, which may be reflected in reduced high-frequency EEG patterns activity (ie, beta and gamma).^13,55,56^

Participants in this study were a subset of the cohort included in a prior study investigating the associations of PAE and PTE with EEG activity at birth.^57^ Based on prior evidence, we hypothesized that the associations observed at birth would persist into early and middle childhood and that distinct patterns of PAE and PTE would be differentially associated with EEG power. Moreover, we hypothesized,^48,57^ that the magnitude of the associations would differ in sex-stratified analyses, such that the brain activity of male participants would be more strongly associated with PAE and PTE.

## Methods

This cohort study was approved by the Avera Research Institute, Columbia University Irving Medical Center, and New York State Psychiatric Institute institutional review boards. Parents or guardians provided written informed consent, and participating children provided assent to participate. The analyses followed the Strengthening the Reporting of Observational Studies in Epidemiology (STROBE) reporting guideline for cohort studies.

### Participants

Participants were originally enrolled in the Safe Passage Study conducted by the Prenatal Alcohol and SIDS and Stillbirth (PASS) Network, a multicenter study investigating the roles of PAE and PTE in risk for multiple adverse outcomes.^58^ In brief, consenting, pregnant people carrying 1 or 2 fetuses, aged 16 years or older, from 6 weeks’ gestation up to but not including the delivery admission, and able to speak English or Afrikaans were eligible to participate in the study. Pregnant people planning to terminate their pregnancy or move out of the catchment area or advised against participation by a health care practitioner were excluded. From December 2011 through August 2015, pregnant individuals were recruited from 2 residential areas within Cape Town, South Africa, and from 5 clinical sites in the Northern Plains (South Dakota and North Dakota), US; only participants in the US were enrolled in the follow-up study.

A subset of the participants originally enrolled in PASS were reenrolled in the Environmental influences on Child Health Outcomes (ECHO) study, conducted in South Dakota at 2 locations (Sioux Falls and Rapid City) and led by the Avera Research Institute. Parents or guardians and children were eligible to participate in the ECHO study if the children were former PASS participants, parents or guardians were able to provide informed consent, and children were able to provide assent. Parents or guardians currently incarcerated, under house arrest, on parole, unable to provide consent, or advised against participation by a health care practitioner were excluded. From September 2018 through November 2022, as part of the ECHO study, children were invited to participate in an EEG study at ages 4, 5, 7, 9, or 11 years. The primary caregivers provided informed consent for themselves and the children to participate in the ECHO study and the EEG portion of the visit (children aged ≥8 years provided assent to participate) (eFigure in Supplement 1). Self-reported information on birth weight, education levels, gestational age at birth, household monthly income, marital status, and race were collected. Race was categorized as American Indian or Alaska Native, White, or other (including Asian, Black or African American, Native Hawaiian or Other Pacific Islander, and other race not specified). Race was included to describe the population included in this cohort study.

### Maternal Self-Reported PAE and PTE Measures

The methods used to collect prenatal exposures have been published elsewhere.^59,60^ Pregnant people completed in-person study visits, including a recruitment visit and up to 3 prenatal visits occurring at 20 to 24, 28 to 32 and 34 or more weeks’ gestation, dependent on gestational age at enrollment. Maternal self-reported alcohol and tobacco cigarette consumptions were captured via a modified version of the Timeline Follow-Back questionnaire attempted at each in-person study visit. After imputation, the data on in utero exposure were used to estimate the number of standard drinks consumed per day and the mean number of cigarettes smoked per week during gestation. A finite mixture model–based approach derived clusters of PAE and PTE^61^; 10 clusters were derived for PAE (nondrinking, high continuous with binging episodes, moderate continuous with binging episodes, low continuous with binging episodes, moderate continuous without binging episodes, low continuous without binging episodes, high quit early with binging episodes, moderate quit early with binging episodes, moderate quit early without binging episodes, and low quit early without binging episodes), and 4 clusters were derived for PTE (nonsmoking, high continuous, low continuous, and quit early). *Quit early* is defined as cessation of exposure by the end of the first trimester, and *continuous* is defined as exposure throughout pregnancy or cessation after the first trimester.

### EEG Data Acquisition and Study Protocol and Processing

EEG brain activity was acquired (vertex-referenced) using a 64-channel HydroCel Geodesic Sensor Net (Magstim EGI). Data were sampled at 500 Hz and stored using the Net Station EGI software version 5.4 (Magstim EGI). Prior to data collection, impedance values for all channels were checked and confirmed to measure less than 50 kΩ. After EEG cap placement, children were seated approximately 70 cm in front of a computer monitor and asked to fixate on a central crosshair. Participants completed a protocol including a total of 3 minutes of alternating 30-second blocks of eyes-open (EO) and eyes-closed (EC) baseline (resting) recording. Instructions were presented in E-Prime version 2.0.10 (Psychology Software Tools). The equipment used and protocols followed during EEG acquisition were identical across ages at assessment and at the 2 data collection sites. EEG data processing and feature extraction procedures are described in the eMethods in Supplement 1. Estimates of periodic EEG activity for the theta, alpha, beta, and gamma frequency bands were extracted for each participant.

### Statistical Analysis

Given the smaller sample size included in this analysis (649 participants in the ECHO cohort) compared with the sample included in the clusters analysis (10 529 participants in the PASS cohort),^61^ the original PAE and PTE clusters were collapsed into 3 PAE and 3 PTE groups. In this study, the PAE clusters were categorized as continuous drinking (by combining high continuous with binging episodes, moderate continuous with binging episodes, low continuous with binging episodes, moderate continuous drinking without binging episodes, and low continuous without binging episodes), quit-early drinking (by combining high quit early with binging episodes, moderate quit early with binging episodes, moderate quit early without binging episodes, and low quit early without binging episodes), and nondrinking and the 3 PTE clusters as continuous smoking (by combining high continuous and low continuous), quit-early smoking, and nonsmoking.

First, separate repeated-measures analyses of variance for each age group were used to verify the stability of the EEG power estimates across the blocks of EO and EC (within-participants terms) (eTable 1 in Supplement 1). For the main analysis, separate generalized linear regression models were used to estimate the associations of PAE (reference: nondrinking) and PTE (reference: nonsmoking) clusters with EEG band power estimates (theta, alpha, beta, and gamma). All models included biological sex, age at EEG assessment (4, 5, 7, 9, or 11 years), number of EEG epochs included in the EEG band power estimates (range, 1 to 30), and maternal education (<high school, completed high school, or >high school) as covariates. Additionally, exploratory sex stratified analyses were performed. The overlap between PAE and PTE clusters was insufficient to examine interactions between exposures in EEG outcomes.

The main analyses were conducted by considering EEG periodic activity collected during blocks of EO and considering EEG periodic activity collected during blocks of EC. An univariable selection method was used to identify the covariates included in these models.^62^ Sensitivity analyses were conducted considering the same models when including a single EEG recording per participant (separately for blocks of EO and blocks of EC) and using generalized estimating equation model clustering at the individual level when retaining all available EEG recordings (separately for blocks of EO and blocks of EC).

Statistical analyses were performed in R Studio version 2023.09.1 + 494 (mounting R version 4.3.1; R Project for Statistical Computing) with deidentified data. The a priori level of significance was set to α = .05, and hypothesis tests were 2-sided. Data were analyzed from November 2022 to November 2023.

## Results

### Summary Demographic Information

The final analysis included 795 usable EEG recordings from 649 participants at ages 4 years (113 participants [17.4%]), 5 years (139 participants [21.4%]), 7 years (194 participants [29.9%]), 9 years (104 participants [16.0%)], or 11 years (99 participants [15.3%]) (Table 1). Most participants were female (333 participants [51.3%]) and the distribution of biological sex was similar across age groups. Stratified by PAE, 334 participants (51.5%) had no PAE, 280 participants (43.1%) had quit-early PAE, and 35 participants (5.4%) had continuous-drinking PAE. Stratified by PTE, 567 participants (87.4%) had no PTE, 33 participants (5.1%) had quit-early PTE, and 49 participants (7.6%) had continuous-smoking PTE. The distribution of PAE was similar in males and females. Conversely, a higher proportion of males were exposed to PTE compared with females. The proportions of PAE and PTE clusters were not different by age groups (eTable 2 and eTable 3 in Supplement 1). The distributions of birth weight, education levels, gestational age at birth, household monthly income, marital status, and race were similar across age and sex groups (Table 1).

**Table 1.** Study Participant Demographic Information

Characteristic	Participants, No. (%)					
	Age 4 y	Age 5 y	Age 7 y	Age 9 y	Age 11 y	Total^a^
Total	113 (17.4)	139 (21.4)	194 (29.9)	104 (16.0)	99 (15.3)	649 (100)
Sex assigned at birth						
Male	60 (53.1)	65 (46.8)	92 (47.4)	52 (50.0)	47 (47.5)	316 (48.7)
Female	53 (46.9)	74 (53.2)	102 (52.6)	52 (50.0)	52 (52.0)	333 (51.3)
Gestational age at birth, mean (SD), wk						
Mean (SD)	38.91 (1.75)	38.94 (1.91)	38.86 (2.17)	38.91 (1.56)	39.15 (1.80)	38.94 (1.90)
<37	8 (7.1)	18 (12.9)	29 (14.9)	8 (7.7)	6 (6.1)	69 (10.6)
≥37	105 (92.9)	121 (87.1)	165 (85.1)	96 (92.3)	93 (93.9)	580 (89.4)
Birth weight, g						
Mean (SD)	3415.20 (630.6)	3416.51 (575.8)	3341.50 (605.5)	3473.86 (552.2)	3469.40 (527.8)	3411.02 (584.7)
<2500	5 (4.4)	10 (7.2)	16 (8.2)	4 (3.8)	4 (4.1)	39 (6.0)
≥2500	108 (95.6)	128 (92.8)	178 (91.8)	100 (96.2)	94 (95.9)	608 (94.0)
Race, No (%)						
American Indian or Alaska Native	16 (14.2)	21 (15.1)	18 (9.3)	10 (9.6)	10 (10.1)	75 (11.6)
White	89 (78.8)	104 (74.8)	161 (83.0)	92 (88.5)	86 (86.9)	532 (82.0)
Other^b^	8 (7.1)	14 (10.1)	15 (7.7)	2 (1.9)	3 (3.0)	42 (6.5)
Maternal characteristics						
PAE cluster						
Nondrinking	59 (52.2)	64 (46.0)	105 (54.1)	50 (48.1)	56 (56.6)	334 (51.5)
Quit-early drinking	44 (38.9)	64 (46.0)	80 (41.2)	49 (47.1)	43 (43.3)	280 (43.1)
Continuous drinking	10 (8.8)	11 (7.9)	9 (4.6)	5 (4.8)	0 (0.0)	35 (5.4)
PTE cluster						
Nonsmoking	93 (82.3)	117 (84.2)	178 (91.8)	91 (87.5)	88 (88.9)	567 (87.4)
Quit-early smoking	6 (5.3)	11 (7.9)	6 (3.1)	6 (5.8)	4 (4.0)	33 (5.1)
Continuous smoking	14 (12.4)	11 (7.9)	10 (5.2)	7 (6.7)	7 (7.1)	49 (7.6)
Education						
<High school	10 (8.8)	12 (8.6)	11 (5.7)	6 (5.8)	4 (4.0)	43 (6.6)
Completed high school	16 (14.2)	14 (10.1)	18 (9.3)	15 (14.4)	7 (7.1)	70 (10.8)
>High school	87 (77.0)	113 (81.3)	165 (85.1)	83 (79.8)	88 (88.9)	536 (82.6)
Monthly income, $						
250-3500	79 (69.9)	107 (62.2)	174 (61.7)	71 (55.0)	57 (57.6)	488 (61.4)
>3500	34 (30.1)	65 (37.8)	105 (37.2)	57 (44.2)	42 (42.4)	303 (38.1)
Marital status						
Single	16 (14.2)	13 (9.4)	20 (10.3)	10 (9.6)	12 (12.1)	71 (10.9)
Married	97 (85.8)	126 (90.6)	174 (89.7)	94 (90.4)	87 (87.9)	578 (89.1)

Abbreviations: PAE, prenatal alcohol exposure; PTE, prenatal tobacco exposure.

^a^ Total counts may not add up to total due to missing covariate data.

^b^ Includes Asian, Black or African American, Native Hawaiian or Other Pacific Islander, other race not specified.

### Stability of EEG Measures Across EO and EC Conditions

Given the stability of the EEG power measures across repetitions of the paradigm (irrespective of the EO-EC condition), maximization of the sample size for analysis was prioritized (eTable 1 in Supplement 1). This design was achieved by solely including the first epoch of either EO or EC in primary analyses. The associations of PAE and PTE clusters with EEG power measures were substantially equivalent across the EO and EC conditions. Here, we present results in the EO condition, given that EEG data collected in this condition have been reported to resembles a true awake resting state paradigm.^63,64^ EC results are presented in eTable 4 and eTable 5 in Supplement 1. The results of the sensitivity analyses, including a single EEG recording per participant, are presented in eTable 6 and eTable 7 in Supplement 1, and results using generalized estimating equation model clustering are presented in eTable 8 and eTable 9 in Supplement 1.

### Associations of PAE Clusters With EEG Power

**Figure 1.**
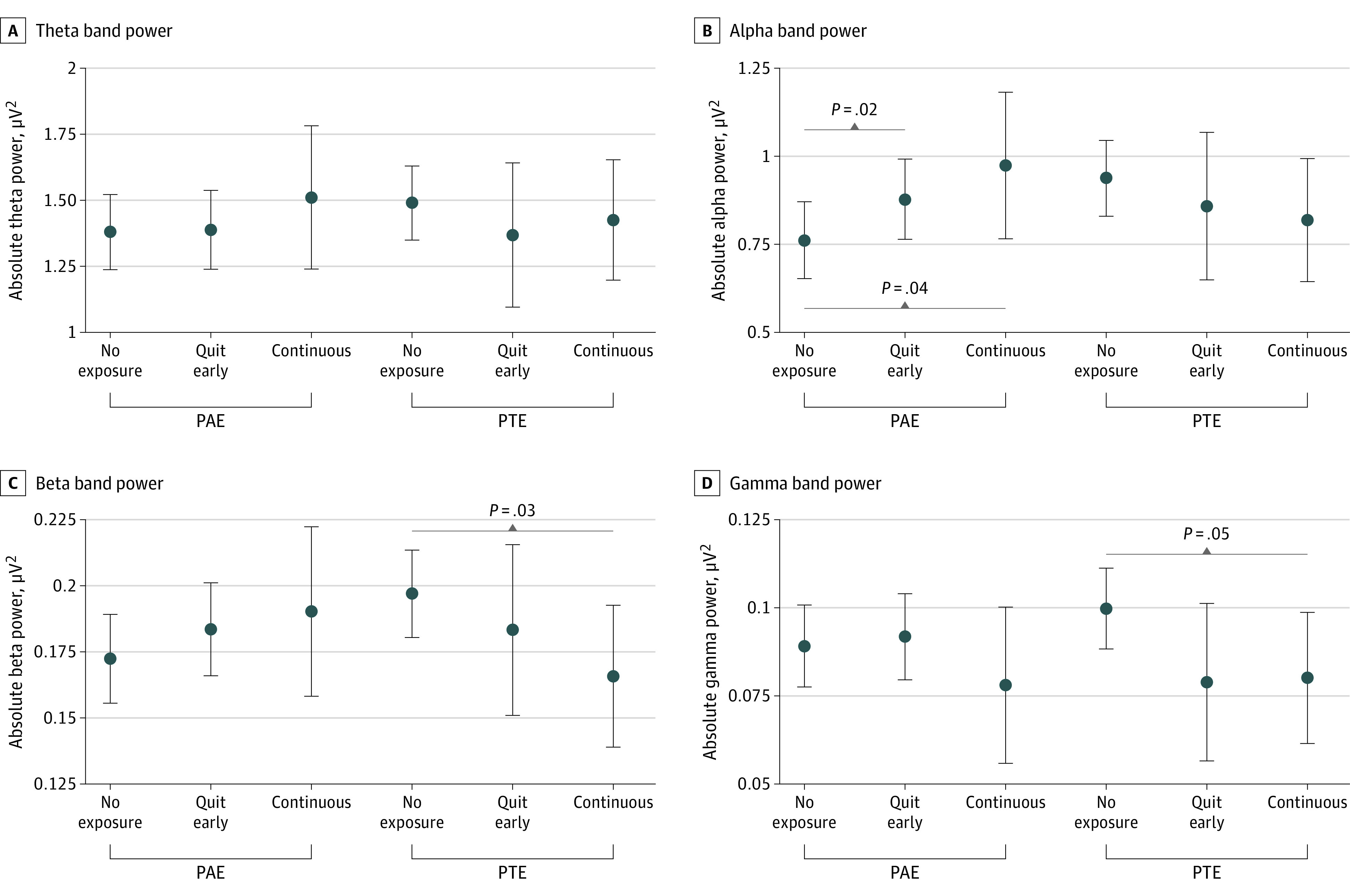
Marginal Means of Electroencephalography Power for the Analyzed Frequency Bands Dots indicate marginal means; whiskers, 95% CIs; PAE, prenatal alcohol exposure; PTE, prenatal tobacco exposure.

There was a significant association of PAE with EEG power in the alpha band (Table 2). Participants whose birthing parents were in the quit-early drinking cluster had an increased alpha power (0.116 [95% CI, 0.023 to 0.209] μV^2^; *P* = .02) compared with individuals without PAE (Table 2 and Figure 1). The magnitude of this increase was approximately double for participants exposed to continuous drinking (0.211 [95% CI, 0.005 to 0.417] μV^2^; *P* = .04) (Table 2 and Figure 1).

**Table 2.** Estimates of the Association of PAE and PTE With EEG Power^a^

Variable	EEG frequency band							
	Theta		Alpha		Beta		Gamma	
	Marginal mean (95% CI), μV^2^	*P* value	Marginal mean (95% CI), μV^2^	*P* value	Marginal mean (95% CI), μV^2^	*P* value	Marginal mean (95% CI), μV^2^	*P* value
PAE (vs nondrinking)								
Quit-early drinking	0.009 (−0.113 to 0.131)	.89	0.116 (0.023 to 0.209)	.02	0.011 (−0.003 to 0.026)	.13	0.003 (−0.007 to 0.013)	.61
Continuous drinking	0.131 (−0.137 to 0.399)	.34	0.211 (0.005 to 0.417)	.04	0.018 (−0.014 to 0.050)	.27	−0.011 (−0.033 to 0.011)	.32
PTE (vs nonsmoking)								
Quit-early smoking	−0.121 (−0.400 to 0.158)	.40	−0.079 (−0.293 to 0.135)	.47	−0.014 (−0.047 to 0.019)	.42	−0.021 (−0.044 to 0.002)	.07
Continuous smoking	−0.064 (−0.299 to 0.171)	.59	−0.118 (−0.299 to 0.062)	.20	−0.031 (−0.059 to −0.003)	.03	−0.020 (−0.039 to −0.000)	.04
Age at EEG assessment, per 1-y increase	−0.138 (−0.166 to −0.110)	<.001	−0.029 (−0.051 to −0.008)	.007	0.000 (−0.003 to 0.004)	.81	−0.004 (−0.007 to −0.002)	<.001
Assigned female sex at birth (vs male)	−0.476 (−0.596 to −0.357)	<.001	−0.237 (−0.329 to −0.146)	<.001	−0.054 (−0.068 to −0.040)	<.001	−0.031 (−0.040 to −0.021)	<.001
Maternal education (vs >high school)								
<High school	−0.016 (−0.275 to 0.242)	.90	0.105 (−0.093 to 0.304)	.30	0.041 (0.010 to 0.072)	<.001	0.044 (0.023 to 0.065)	<.001
Completed high school	−0.018 (−0.210 to 0.175)	.86	−0.007 (−0.155 to 0.141)	.93	−0.002 (−0.025 to 0.021)	.88	0.004 (−0.012 to 0.019)	.66
EEG epoch analyzed, per 1-unit increase^b^	−0.016 (−0.026 to −0.006)	.002	0.003 (−0.005 to 0.011)	.41	−0.002 (−0.003 to −0.001)	.003	−0.002 (−0.003 to −0.001)	<.001

Abbreviations: EEG, electroencephalography; PAE, prenatal alcohol exposure; PTE, prenatal tobacco exposure.

^a^ Includes only eyes-open EEG data. Eyes-closed EEG data are provided in eTable 4 in Supplement 1.

^b^ Reference: 30 epochs.

**Figure 2.**
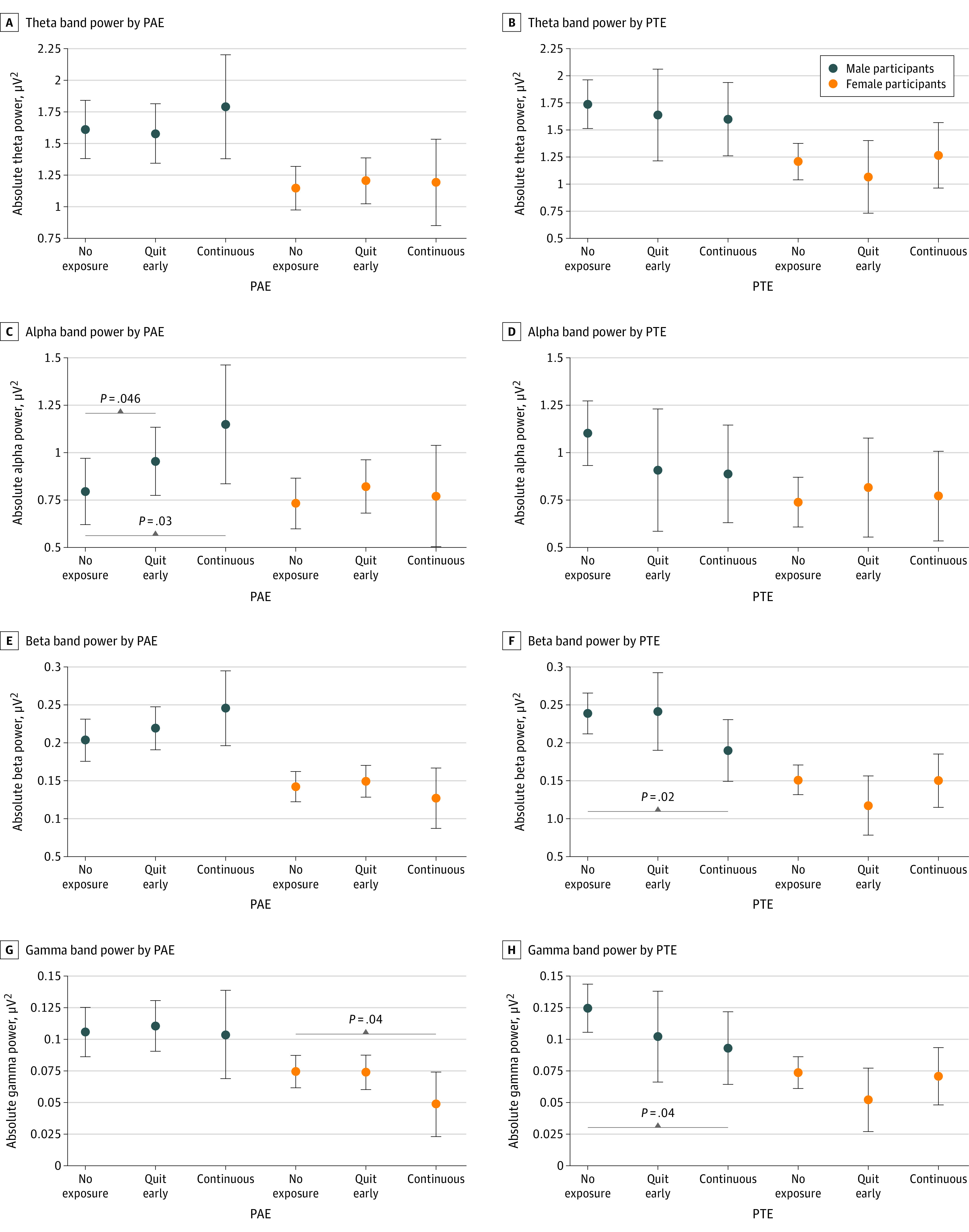
Marginal Means of Electroencephalography Power for the Analyzed Frequency Bands Dots indicate marginal means; whiskers, 95% CIs; PAE, prenatal alcohol exposure; PTE, prenatal tobacco exposure.

In exploratory sex-stratified analyses, this association was only found in males. Male participants in the quit-early PAE cluster had greater EEG power in the alpha band (0.159 [95% CI, 0.003 to 0.315] μV^2^; *P* = .04) compared with individuals without PAE (Table 3 and Figure 2). The magnitude of such increase was approximately double for male participants with continuous PAE (0.354 [95% CI, 0.041 to 0.667] μV^2^; *P* = .03). There were no significant associations of PAE with EEG power in the theta or beta bands. There was an association between PAE with gamma power in females (−0.026 [95% CI, −0.051 to − 0.001] μV^2^; *P* = .04).

**Table 3.** Estimates of the Association of PAE and PTE With EEG Power in Sex-Stratified Analyses

Variable	EEG frequency band^a^								
	Theta		Alpha		Beta			Gamma	
	Marginal mean (95% CI), μV^2^	*P* value	Marginal mean (95% CI), μV^2^	*P* value	Marginal mean (95% CI), μV^2^	*P* value	Marginal mean (95% CI), μV^2^		*P* value
**Males**									
PAE (vs non-drinking)									
Quit-early drinking	−0.033 (−0.238 to 0.172)	.75	0.159 (0.003 to 0.315)	.04	0.016 (−0.009 to 0.040)	.20	0.005 (−0.013 to 0.022)		.59
Continuous drinking	0.180 (−0.231 to 0.591)	.39	0.354 (0.041 to 0.667)	.03	0.042 (−0.007 to 0.091)	.10	−0.002 (−0.037 to 0.033)		.90
PTE (vs nonsmoking)									
Quit-early smoking	−0.099 (−0.534 to 0.336)	.65	−0.195 (−0.526 to 0.136)	.25	0.003 (−0.050 to 0.055)	.92	−0.022 (−0.059 to 0.015)		.24
Continuous smoking	−0.139 (−0.487 to 0.210)	.43	−0.215 (−0.480 to 0.050)	.11	−0.048 (−0.090 to −0.007)	.02	−0.032 (−0.061 to −0.002)		.04
Age at EEG assessment, per 1-y increase	−0.176 (−0.224 to −0.127)	<.001	−0.021 (−0.057 to 0.016)	.27	0.001 (−0.005 to 0.007)	.76	−0.005 (−0.010 to −0.001)		.009
Maternal education (vs >high school)									
<High school	−0.054 (−0.463 to 0.355)	.80	0.194 (−0.117 to 0.506)	.22	0.060 (0.011 to 0.109)	.02	0.048 (0.014 to 0.083)		.007
Completed high school	−0.105 (−0.462 to 0.253)	.57	−0.101 (−0.373 to 0.172)	.47	0.003 (−0.040 to 0.046)	.90	0.016 (−0.015 to 0.046)		.31
EEG epoch analyzed, per 1-unit increase^b^	−0.010 (−0.026 to 0.005)	.19	0.005 (−0.007 to 0.016)	.45	−0.001 (−0.003 to 0.000)	.14	−0.003 (−0.004 to −0.001)		<.001
**Females**									
PAE (vs nondrinking)									
Quit-early drinking	0.058 (−0.079 to 0.196)	.41	0.089 (−0.018 to 0.197)	.10	0.007 (−0.009 to 0.023)	.37	−0.001 (−0.011 to 0.010)		.89
Continuous drinking	0.046 (−0.292 to 0.384)	.79	0.039 (−0.224 to 0.303)	.77	−0.015 (−0.054 to 0.024)	.45	−0.026 (−0.051 to −0.001)		.04
PTE (vs nonsmoking)									
Quit-early smoking	−0.141 (−0.482 to 0.201)	.42	0.077 (−0.190 to 0.344)	.57	−0.034 (−0.073 to 0.006)	.09	−0.022 (−0.047 to 0.004)		.10
Continuous smoking	0.058 (−0.254 to 0.370)	.72	0.032 (−0.212 to 0.276)	.80	−0.001 (−0.037 to 0.035)	.95	−0.003 (−0.026 to −0.020)		.81
Age at EEG assessment, per 1-y increase	−0.105 (−0.137 to −0.074)	<.001	−0.037 (−0.061 to −0.012)	.003	−0.000 (−0.004 to 0.004)	.98	−0.003 (−0.006 to −0.001)		.004
Maternal education (vs >high school)									
<High school	−0.016 (−0.329 to 0.297)	.92	0.016 (−0.228 to 0.260)	.90	0.020 (−0.017 to 0.056)	.29	0.038 (0.014 to 0.061)		.002
Completed high school	0.056 (−0.148 to 0.261)	.59	0.025 (−0.135 to 0.185)	.76	−0.009 (−0.033 to 0.014)	.43	−0.007 (−0.022 to 0.0408)		.36
EEG epoch analyzed, per 1-unit increase^b^	−0.022 (−0.035 to −0.009)	<.001	−0.002 (−0.012 to 0.008)	.72	−0.003 (−0.004 to −0.001)	<.001	−0.001 (−0.002 to 0.000)		.06

Abbreviations: EEG, electroencephalography; PAE, prenatal alcohol exposure; PTE, prenatal tobacco exposure.

^a^ Includes only eyes-open EEG data. Eyes-closed EEG data are provided in eTable 5 in Supplement 1.

^b^ Reference: 30 epochs.

### Associations of PTE Clusters With EEG Power

PTE was associated with decreased EEG power in the beta and gamma bands. Participants whose birthing parents were in the continuous PTE cluster had a decrease in beta power (−0.031 [95% CI, −0.059 to −0.003] μV^2^; *P* = .03) compared with participants without PTE. Compared with the nonsmoking cluster, male participants whose birthing parents were in the continuous PTE cluster had a decrease in beta power (−0.048 [95% CI, −0.090 to −0.007] μV^2^; *P* = .02) (Table 3 and Figure 2). Participants with continuous PTE had decreased EEG power in the gamma band (−0.020 [95% CI, −0.039 to −0.000] μV^2^; *P* = .04) (Table 3 and Figure 1). In exploratory sex-stratified analyses, the association between PTE and EEG power in the gamma band (in the overall models) remained significant for male participants only: male participants with continuous PTE had decreased gamma power (−0.032 [95% CI, −0.061 to −0.002] μV^2^; *P* = .04) (Table 3 and Figure 2). There was not a significant association of PTE with EEG theta power or alpha power.

## Discussion

This cohort study is the largest study to our knowledge to investigate the associations of PAE and PTE with EEG power in early and middle childhood. Long-term associations were observed across EEG frequency bands and were differentially expressed in males vs females. Specifically, PAE was predominantly associated with EEG in the lower frequency bands (alpha), whereas associations with PTE were reported for the higher frequency bands (beta and gamma). PAE was associated with increased alpha EEG power in a dose-dependent association, such that individuals with PAE after the first trimester had the most significant increase compared with participants without PAE. Moreover, termination of alcohol consumption in the first trimester (quit-early group) was associated with alterations in EEG power in the alpha band. Conversely, associations of PTE with EEG power were reported in the subset of participants exposed continuously to tobacco smoking during pregnancy, such that EEG power measures in the beta and gamma bands were lower than those in the unexposed group.

These findings are in agreement with and extend our previous results obtained in a subset of this cohort (163 participants) whose EEG recordings were acquired at birth during sleep.^57^ We found PAE to be similarly associated with an increase in alpha power. However, no association of PAE with theta power was found in early and middle childhood. Similarly for the higher frequency ranges, the associations of PTE with beta and gamma bands persisted after birth. Specifically, participants exposed to PTE after the first trimester displayed a similar decrease in EEG power in early and middle childhood. Taken together, our results support long-term associations of PAE and PTE with oscillatory brain activity. Profiles of increases in low-frequency EEG power and decreases in high-frequency EEG power have been associated with heightened risk for neurodevelopmental disorders.^65,66,67,68,69^ In animal models, such profiles of altered brain cortical activity are thought to reflect an imbalance in the excitatory-inhibitory attributable to alterations in the GABAergic interneuron differentiation and migration.^70^ Moreover, PAE and PTE have been reported to alter hippocampal microglia polarization and promote inflammatory signaling leading to long-term disruption of the brain neurosignaling.^71,72^ As the primary immune cells of the brain, microglia are extremely sensitive to perturbations and thus have the capacity to alter brain development trajectory in a sex-dependent manner.^73^ Interestingly, we observed pronounced sex differences in EEG power in early and middle childhood. Specifically, most of the associations observed in overall models were only found in males. This increased male-specific vulnerability may reflect the heightened underlying susceptibility of male individuals with neurodivergent phenotypes to PAE and PTE; which could be partially explain the higher prevalence of diagnoses of neurodevelopmental conditions in males.^74,75,76,77^ Understanding the role of sex differences in brain development opens the possibility to study pathways of vulnerability or resilience.^78^

Our prior findings, combined with those from this study, suggest that any level of PAE or PTE has long-term associations with brain activity from birth through early and middle childhood, strengthening the notion that research has not yet determined a safe level of alcohol or tobacco use during pregnancy.^46,47,48,49^ Moreover, to our knowledge, we are the first to report long-term associations between PAE and brain activity even in children with low PAE and whose mothers quit drinking before the second trimester. Similar conclusions can be drawn for PTE. These levels of PAE and PTE are commonly reported by pregnant individuals in the US and globally; hence, it is imperative to underscore the public health relevance of these results in the context of media reports on the lack of perinatal effects from light drinking and smoking during pregnancy.^22,23^ As a final point, our results should not imply that PAE or PTE lead to permanent alterations of functional brain development. Many factors can influence brain development trajectories, including a variety of intervention targets and mechanisms for resilience.

### Limitations

This study has limitations that warrant consideration. First, the determination of PAE and PTE statuses relied on self-reported data, which could have resulted in underreporting or overreporting. However, any misclassification would probably be comparable across the analyzed exposure clusters, thereby reducing the precision of the models and biasing the estimates toward the null. Moreover, underreporting, which is the most likely bias in self-reported data, would add to uncertainty of exposures, which might mask significant findings but should not contribute to findings that were significant. Additionally, our analyses did not consider the potential impact of exposure to passive smoking in either the prenatal or postnatal periods. Residual confounding attributable to illicit drug use or other unmeasured postnatal variables is likely. Given the small numbers of participants belonging to the continuous PAE and PTE groups and the insufficient overlap between PAE and PTE clusters, this study was not powered to examine interactions between drinking and smoking in EEG power. Moreover, EEG power was not analyzed at the regional level but rather by calculating the mean power across brain regions, as the former approach would have not provided a sufficient sample size for statistical analysis. Furthermore, the number of participants with multiple EEG recordings was insufficient for conducting any longitudinal trajectory analysis. Moreover, the cross-sectional nature of the cohort is not adequate for evaluating the persistence, nor washout effect of the findings.

## Conclusions

The findings of this cohort study suggest that even low levels of PAE and PTE were associated with long-term alterations of brain activity. Features derived from EEG signals have potential as reliable indicators and sensitive biomarkers of the long-term associations of PAE and PTE with functional brain activity. Given the widespread levels of drinking and smoking behaviors in pregnant people, our findings have public health implications at the population level. These results are likely generalizable to children living in low- and mid-income communities and settings in the US and worldwide whose mothers were exposed to low to moderate amounts of tobacco or alcohol. Further efforts are needed to identify the contributing role, either decremental or protective, of a variety of postnatal exposures and environmental factors on the investigated associations.

## Notes

Supplement 1.
eMethods.
**eTable 1.** *F*-Statistic of Repeated Measures ANOVAs **eTable 2.** Crosstabulation of the Distributions of PAE and PTE by Age at EEG Assessment **eTable 3.** Crosstabulation of the Joint Distribution of PAE and PTE **eTable 4.** Estimates of the Association of PAE and PTE With EEG Power in EC Blocks **eTable 5.** Estimates of the Association of PAE and PTE With EEG Power in EC Blocks in Sex-Stratified Analyses **eTable 6.** Estimates of the Associations of PAE and PTE With EEG Power in EO Blocks in Sensitivity Analysis No. 1 **eTable 7.** Estimates of the Associations of PAE and PTE With EEG Power in EC Blocks in Sensitivity Analysis No. 1 **eTable 8.** Estimates of the Associations of PAE and PTE With EEG Power in EO Blocks in Sensitivity Analysis No. 2 **eTable 9.** Estimates of the Associations of PAE and PTE With EEG Power in EC Blocks in Sensitivity Analysis No. 2 **eFigure.** Participant Recruitment Flowchart
eReferences.

Supplement 2.
Data Sharing Statement
