# Supplementary material for: In Utero Exposure to Alcohol and Tobacco and Electroencephalogram Power During Childhood
Source: JAMA Netw Open. 2024 Jan 5;7(1):e2350528. doi: 10.1001/jamanetworkopen.2023.50528 (PMC10770777; doi:10.1001/jamanetworkopen.2023.50528)
Supplement: Supplement 1. — eMethods. eTable 1. F-Statistic of Repeated Measures ANOVAs eTable 2. Crosstabulation of the Distributions of PAE and PTE by Age at EEG Assessment eTable 3. Crosstabulation of the Joint Distribution of PAE and PTE eTable 4. Estimates of the Association of PAE and PTE With EEG Power in EC Blocks eTable 5. Estimates of the Association of PAE and PTE With EEG Power in EC Blocks in Sex-Stratified Analyses eTable 6. Estimates of the Associations of PAE and PTE With EEG Power in EO Blocks in Sensitivity Analysis No. 1 eTable 7. Estimates of the Associations of PAE and PTE With EEG Power in EC Blocks in Sensitivity Analysis No. 1 eTable 8. Estimates of the Associations of PAE and PTE With EEG Power in EO Blocks in Sensitivity Analysis No. 2 eTable 9. Estimates of the Associations of PAE and PTE With EEG Power in EC Blocks in Sensitivity Analysis No. 2 eFigure. Participant Recruitment Flowchart eReferences. [file jamanetwopen-e2350528-s001.pdf]

Supplemental Online Content

Pini N, Sania A, Rao S, et al. In utero exposure to alcohol and tobacco and electroencephalogram power during childhood. *JAMA Netw Open*. 2024;7(1):e2350528.
doi:10.1001/jamanetworkopen.2023.50528

eMethods.
eTable 1. *F*-Statistic of Repeated Measures ANOVAs
eTable 2. Crosstabulation of the Distributions of PAE and PTE by Age at EEG Assessment
eTable 3. Crosstabulation of the Joint Distribution of PAE and PTE
eTable 4. Estimates of the Association of PAE and PTE With EEG Power in EC Blocks
eTable 5. Estimates of the Association of PAE and PTE With EEG Power in EC Blocks in Sex-Stratified Analyses
eTable 6. Estimates of the Associations of PAE and PTE With EEG Power in EO Blocks in Sensitivity Analysis No. 1
eTable 7. Estimates of the Associations of PAE and PTE With EEG Power in EC Blocks in Sensitivity Analysis No. 1
eTable 8. Estimates of the Associations of PAE and PTE With EEG Power in EO Blocks in Sensitivity Analysis No. 2
eTable 9. Estimates of the Associations of PAE and PTE With EEG Power in EC Blocks in Sensitivity Analysis No. 2
eFigure. Participant Recruitment Flowchart
eReferences.

This supplemental material has been provided by the authors to give readers additional information about their work.

© 2024 Pini N et al. *JAMA Network Open.*

eMethods.

EEG Data Preprocessing and Feature Extraction

EEG data were preprocessed following procedures described in the Maryland Analysis of Developmental EEG (MADE) pipeline (https://github.com/ChildDevLab/MADE-EEG-preprocessing-pipeline).[1] EEG data were high-pass filtered offline at 0.3 Hz and low-pass filtered at 49 Hz. Bad channels were identified and removed using the EEGLAB[2] plug-in FASTER.[3] Ocular artifacts were removed via independent component analysis (ICA) performed on a 1 Hz high-pass filtered copy of the original dataset. Prior to ICA, the copied dataset was segmented into 1 second epochs. Then, noisy segments likely corrupted with muscle artifacts were rejected using a combination of criteria; voltage threshold of ± 1000 μV and spectral threshold (range [-100 dB +30 dB]) within the 20–40 Hz frequency band. In the instance of a channel with greater than 20% of the epochs labelled as noisy, it was removed from both the ICA copied dataset and the original dataset. Then, ICA decomposition was run on the copied dataset and the resulting ICA weights copied back onto the original dataset. Artifactual IC components were removed by using the Adjusted-ADJUST algorithm.[4,5] EEG data were segmented into 2-second epochs and underwent two final steps of artifact rejection. Firstly, to further remove the presence of residual ocular activity, epochs in the ocular channels (1, 5, 10, and 17) with a voltage exceeding ±150 μV were removed. Secondly, any epoch (in non-ocular channels) exceeding ±125 μV was interpolated. Additionally, if the latter condition occurred in more than 10% of the channels (percentage computed not including globally rejected channels) such epoch was removed for all the leads. Lastly, any remaining missing channels were then interpolated using the spherical spline method[6] and data were re-referenced to the average reference.[7]

EEG power spectra were estimated from 1 to 49 Hz using Welch's method with a Hamming window (50% overlap) and a resulting frequency resolution of 0.5 Hz. The obtained EEG spectra were then averaged across all electrodes to compute a single power spectrum for each condition (EO and EC). The specparam methodology (version 1.0.0) was used to parameterize EEG power spectra. Algorithm settings were set as: peak width limits: [1 6]; max number of peaks: 6; minimum peak height: 0.1; peak threshold: 0.5; and aperiodic mode: fixed.[8] Power spectra were parameterized across the frequency range 3 to 40 Hz.[8,9] In brief, this method models a given EEG power spectrum as a linear combination of aperiodic components (in log-log space) and periodic activity (oscillations superimposed the aperiodic signal). The aperiodic activity is fitted to the power spectrum and subsequently removed to enhance the EEG periodic peaks. The detected peaks are iteratively fit and then removed. Once these fitted oscillatory peaks are removed from the power spectrum, a second aperiodic fit is applied to the data. Lastly, goodness of fit measures are computed on the fitted components (aperiodic and periodic activity). For each participant, estimates of periodic activity for the theta (frequency range [3 6] Hz), alpha (frequency range [6.5 13] Hz), beta (frequency range [13.5 30] Hz), and gamma (frequency range [30.5 40] Hz) frequency bands were extracted. The identified lower and upper limits of the frequency ranges were informed by previous results obtained by our group.[10]

Stability of the EEG Measures across EO and EC Blocks

The data collection protocol required participant to be seated ~70 cm in front of a computer monitor and asked to fixate on a central crosshair. Individuals completed a protocol consisting of a total of 3-minutes of alternating 30-s blocks of eyes open (EO) and eyes closed (EC) baseline (resting) recording.

The analysis presented in the following, investigates the distributions of the EEG power measures (theta, alpha, beta, and gamma) across the EO and EC blocks. EEG power estimates in EO, EC blocks, and age groups were analyzed separately given the marked differences reported in previous work by our research groups.[10,11] Data was natural log-transformed. Separate repeated-measures ANOVAs were utilized to verify the stability of the EEG power estimates across the blocks of EO and EC (*within-subjects terms*). Given the numerous tests performed, a 10% false discovery rate (FDR) correction was implemented to correct for multiple comparisons using the Benjamini-Hochberg procedure. In the instance of a significant main effect of either EO or EC blocks, paired comparisons were tested to assess the potential differences between EO1 vs EO2, EO1 vs EO3, and EO2 vs EO3, or EC1 vs EC2, EC1 vs EC3, and EC2 vs EC3. In this case, the Bonferroni correction was used. There was a statistically significant association of EO and EC blocks on EEG power in 11 (27.5%) out of the 40 combinations of ages and frequency bands tested; with a predominance of significant associations found in EO (8 (40.0%) out of 20) compared to EC (3 (15.0%) out of 20). Approximately half (19 (57.6%) out of 33) of the pairwise comparisons (EO1 vs EO2, EO1 vs EO3, and EO2 vs EO3, or EC1 vs EC2, EC1 vs EC3, and EC2 vs EC3) were significant. Moreover, a larger proportion of such pairwise

© 2024 Pini N et al. *JAMA Network Open.*

differences were significant when comparing epoch 1 versus epoch 2 (9 (47.4%)); epoch 1 versus epoch 3 (7 (36.8%)) and epoch 2 versus epoch 3 (3 (15.8%)). Participants in the 7- and 9-years-of-age groups accounted for the majority of these differences. Results are reported in **eTable 1**.

© 2024 Pini N et al. *JAMA Network Open.*

eTable 1. *F*-statistic of repeated measures ANOVAs. Estimates (marginal means (CI) and *p*-values) of the pairwise comparisons are reported for significant models only

Age at EEG	EEG Band	Overall	EO1 vs EO2	EO1 vs EO3	EO2 vs EO3	Overall	EC1 vs EC2	EC1 vs EC3	EC2 vs EC3
4Y	**Theta**	*F*(1.86, 187.94) = 3.63<br>*p*>.05	N/A	N/A	N/A	*F*(2, 198) = 3.66<br>*p*>.05	N/A	N/A	N/A
	**Alpha**	*F*(1.79, 180.43) = 5.56<br>*p*>.05	N/A	N/A	N/A	*F*(1.82, 180.36) = 0.18<br>*p*>.05	N/A	N/A	N/A
	**Beta**	*F*(1.57, 158.51) = 0.45<br>*p*>.05	N/A	N/A	N/A	*F*(2, 198) = 5.79<br>*p*>.05	N/A	N/A	N/A
	**Gamma**	*F*(1.74, 175.3) = 3.79<br>*p*>.05	N/A	N/A	N/A	*F*(2, 198) = 7.19<br>*p*>.05	N/A	N/A	N/A
5Y	**Theta**	*F*(2, 308) = 5.27<br>*p*>.05	N/A	N/A	N/A	*F*(1.84, 282.81) = 0.67<br>*p*>.05	N/A	N/A	N/A
	**Alpha**	*F*(2, 308) = 4.16<br>*p*>.05	N/A	N/A	N/A	*F*(1.91, 293.82) = 0.07<br>*p*>.05	N/A	N/A	N/A
	**Beta**	***F*(1.89, 290.58) = 6.40**<br>***p*<.05**	0.02<br>(-0.01 0.05)<br>*p*>.05	-0.04<br>(-0.08 0.01)<br>*p*>.05	**-0.06**<br>**(-0.09 -0.03)**<br>***p*<.001**	***F*(2, 308) = 13.95**<br>***p*<.05**	**-0.05**<br>**(-0.07 -0.02)**<br>***p*<.001**	**-0.07**<br>**(-0.09 -0.04)**<br>***p*<.001**	-0.03<br>(-0.05 0.00)<br>*p*>.05
	**Gamma**	***F*(1.91, 294.57) = 6.51**<br>***p*<.05**	-0.04<br>(-0.01 0.04)<br>*p*>.05	**-0.14**<br>**(-0.22 -0.06)**<br>***p*<.01**	**-0.09**<br>**(-0.16 -0.03)**<br>***p*<.05**	***F*(1.89, 290.58) = 6.40**<br>***p*<.05**	**-0.13**<br>**(-0.20 -0.05)**<br>***p*<.001**	**-0.19**<br>**(-0.25 -0.11)**<br>***p*<.001**	-0.06<br>(-0.13 0.01)<br>*p*>.05
7Y	**Theta**	***F*(1.79, 488.79) = 26.7**<br>***p*<.001**	**-0.07**<br>**(-0.09 -0.04)**<br>***p*<.001**	**-0.08**<br>**(-0.11 -0.06)**<br>***p*<.001**	-0.02<br>(-0.04 0.00)<br>*p*>.05	*F*(2, 544) = 0.83<br>*p*>.05	N/A	N/A	N/A
	**Alpha**	***F*(1.89, 290.58) = 6.4**<br>***p*<.001**	**0.06**<br>**(0.03 0.09)**<br>***p*<.001**	0.01<br>(-0.08 0.44)<br>*p*>.05	**-0.05**<br>**(-0.07 -0.03)**<br>***p*<.001**	*F*(2, 544) = 0.76<br>*p*>.05	N/A	N/A	N/A
	**Beta**	***F*(1.89, 290.58) = 6.4**<br>***p*<.001**	**0.05**<br>**(0.02 0.07)**<br>***p*<.001**	0.03<br>(-0.00 0.05)<br>*p*>.05	-0.02<br>(-0.04 0.00)<br>*p*>.05	*F*(191, 520.13) = 0.70<br>*p*>.05			
	**Gamma**	*F*(1.89, 515.83) = 1.43	N/A	N/A	N/A	*F*(2, 544) = 4.53	N/A	N/A	N/A

© 2024 Pini N et al. *JAMA Network Open.*

		$p>.05$				$p>.05$			
9Y	**Theta**	**$F(1.89, 290.58)$<br>$= 6.4$<br>$p<.001$**	**-0.09<br>(-0.12 -0.04)<br>$p<.001$**	**-0.09<br>(-0.12 -0.05)<br>$p<.001$**	-0.00<br>(-0.03 0.03)<br>$p>.05$	$F(1.85, 236.36)$<br>$= 2.30$<br>$p>.05$	N/A	N/A	N/A
	**Alpha**	$F(1.62, 206.86)$<br>$= 1.05$<br>$p>.05$	N/A	N/A	N/A	$F(1.77, 226.40)$<br>$= 0.42$<br>$p>.05$	N/A	N/A	N/A
	**Beta**	**$F(1.89, 290.58)$<br>$= 6.4$<br>$p<.01$**	**0.06<br>(0.02 0.10)<br>$p<.01$**	**0.08<br>(0.04 0.11)<br>$p<.001$**	0.02<br>(-0.00 0.04)<br>$p>.05$	**$F(1.83, 234.78)$<br>$= 3.51$<br>$p<.05$**	**0.03<br>(0.21 0.05)<br>$p<.01$**	0.02<br>(-0.00 0.05)<br>$p>.05$	-0.01<br>(-0.04 0.01)<br>$p>.05$
	**Gamma**	$F(1.88, 240.04)$<br>$= 5.58$<br>$p>.05$	N/A	N/A	N/A	$F(1.85, 236.67)$<br>$= 0.92$<br>$p>.05$	N/A	N/A	N/A
11Y	**Theta**	**$F(1.89, 290.58)$<br>$= 6.4$<br>$p<.01$**	**-0.08<br>(-0.16 -0.05)<br>$p<.001$**	**-0.13<br>(-0.16 -0.08)<br>$p<.001$**	-0.04<br>(-0.08 0.00)<br>$p>.05$	$F(1.80, 174.83)$<br>$= 1.25$<br>$p>.05$	N/A	N/A	N/A
	**Alpha**	$F(1.68, 165.08)$<br>$= 1.89$<br>$p>.05$	N/A	N/A	N/A	$F(1.81, 175.23)$<br>$= 0.16$<br>$p>.05$	N/A	N/A	N/A
	**Beta**	$F(1.83, 179.52)$<br>$= 1.62$<br>$p>.05$	N/A	N/A	N/A	$F(1.80, 174.36)$<br>$= 2.33$<br>$p>.05$	N/A	N/A	N/A
	**Gamma**	$F(1.80, 176.81)$<br>$= 2.82$<br>$p>.05$	N/A	N/A	N/A	$F(1.81, 175.4)$<br>$= 2.19$<br>$p>.05$	N/A	N/A	N/A

© 2024 Pini N et al. *JAMA Network Open.*

eTable 2. Crosstabulation of the distributions of PAE and PTE by age at EEG assessment

Participant Characteristics	**4Y** *N=113 (17.4%)*	**5Y** *N=139 (21.4%)*	**7Y** *N=194 (29.9%)*	**9Y** *N=104 (16.0%)*	**11Y** *N=99 (15.3%)*	**Entire Cohort** *N=649 (100.0%)*
**PAE Clusters; No (%)**						
Non-drinking	59 (52.2%)	64 (46.0%)	105 (54.1%)	50 (48.1%)	56 (56.6%)	334 (51.5%)
Quit-early drinking	44 (38.9%)	64 (46.0%)	80 (41.2%)	49 (47.1%)	43 (43.3%)	280 (43.1%)
Continuous drinking	10 (8.8%)	11 (7.9%)	9 (4.6%)	5 (4.8%)	0 (0.0%)	35 (5.4%)
**PTE Clusters; No (%)**						
Non-smoking	93 (82.3%)	117 (84.2%)	178 (91.8%)	91 (87.5%)	88 (88.9%)	567 (87.4%)
Quit-early smoking	6 (5.3%)	11 (7.9%)	6 (3.1%)	6 (5.8%)	4 (4.0%)	33 (5.1%)
Continuous smoking	14 (12.4%)	11 (7.9%)	10 (5.2%)	7 (6.7%)	7 (7.1%)	49 (7.6%)

© 2024 Pini N et al. *JAMA Network Open.*

eTable 3. Crosstabulation of the joint distribution of PAE and PTE

	PTE Clusters; No (%)		
**PAE Clusters; No (%)**	Non-smoking	Quit-early smoking	Continuous smoking
Non-drinking	300 (46.20%)	14 (2.15%)	20 (3.10%)
Quit-early drinking	244 (37.60%)	14 (2.15%)	22 (3.40%)
Continuous drinking	23 (3.50%)	5 (0.80%)	7 (1.10%)

© 2024 Pini N et al. *JAMA Network Open.*

Associations of PAE clusters on EEG Power, Associations of PTE clusters on EEG Power, Associations of Covariates on EEG Power

eTable 4. Estimates (marginal means (CI) and *p*-values) of the association of PAE and PTE and EEG power (EC blocks only)

	**EEG Frequency Bands**							
Predictors	**Theta (μV^2)**		**Alpha (μV^2)**		**Beta (μV^2)**		**Gamma (μV^2)**	
**PAE [reference: non-drinking]**								
Quit-early drinking	-0.170 (-0.394 0.053)	> .05	**0.203 (0.027 0.379)**	**< .01**	-0.008 (-0.007 0.023)	> .05	-0.003 (-0.011 0.005)	> .05
Continuous drinking	0.173 (-0.322 0.668)	> .05	**0.385 (0.005 0.775)**	**< .05**	**0.038 (0.005 0.071)**	**< .01**	-0.009 (-0.027 0.009)	> .05
**PTE [reference: non-smoking]**								
Quit-early smoking	0.144 (-0.317 0.660)	> .05	-0.002 (-0.385 0.428)	> .05	-0.003 (-0.037 0.031)	> .05	-0.006 (-0.024 0.012)	> .05
Continuous smoking	-0.313 (-0.747 0.121)	> .05	-0.309 (-0.651 0.003)	> .05	-0.014 (-0.043 0.014)	> .05	0.012 (-0.003 0.028)	> .05
**Age at EEG assessment**	**-0.236 (-0.288 -0.184)**	**< .001**	**0.044 (0.003 0.085)**	**< .05**	**0.006 (0.003 0.010)**	**< .001**	**-0.003 (-0.005 -0.001)**	**< .001**
**Sex assignment at birth [reference: male]**	**-0.779 (-0.998 -0.448)**	**< .001**	**-0.567 (-0.740 -0.395)**	**< .001**	**-0.050 (-0.065 -0.036)**	**< .001**	**-0.024 (-0.032 -0.016)**	**< .05**
**Maternal Education [reference: beyond high school]**								
Less than high school	-0.036 (-0.519 0.446)	> .05	0.124 (-0.256 0.504)	> .05	0.024 (-0.008 0.056)	> .05	**0.022 (0.005 0.039)**	**< .001**
Completed high school	0.096 (-0.256 0.448)	> .05	-0.128 (-0.406 0.149)	> .05	-0.003 (-0.026 0.020)	> .05	0.006 (-0.007 0.018)	> .05
EEG epoch analyzed	-0.007 (-0.023 0.010)	> .05	0.011 (-0.256 0.504)	> .05	-0.001 (-0.002 0.000)	> .05	**-0.001 (-0.001 -0.000)**	**< .05**

© 2024 Pini N et al. *JAMA Network Open.*

eTable 5. Estimates (marginal means (CI) and *p*-values) of the association of PAE and PTE and EEG power (EC blocks only) in sex-stratified analyses (models only including male participants (top) or female participants (bottom))

Males	**EEG Frequency Bands**							
Predictors	**Theta (µV²)**		**Alpha (µV²)**		**Beta (µV²)**		**Gamma (µV²)**	
**PAE [reference: non-drinking]**								
Quit-early drinking	-0.369 (-0.740 0.002)	> .05	0.244 (-0.043 0.532)	> .05	0.008(-0.016 0.033)	> .05	-0.005 (-0.019 0.009)	> .05
Continuous drinking	0.328 (-0.427 1.082)	> .05	**0.761 (0.175 1.347)**	**< .05**	**0.073 (0.023 0.122)**	**< .01**	-0.005 (-0.034 0.023)	> .05
**PTE [reference: non-smoking]**								
Quit-early smoking	-0.199 (-0.986 0.589)	> .05	-0.234 (-0.845 0.377)	> .05	0.015 (-0.036 0.067)	> .05	0.005 (-0.025 0.035)	> .05
Continuous smoking	-0.600 (-1.242 0.042)	> .05	-0.471 (-0.970 0.027)	> .05	-0.025 (-0.067 0.017)	> .05	0.005 (-0.020 0.029)	> .05
**Age at EEG assessment**	**-0.298 (-0.385 -0.212)**	**< .001**	**0.076 (0.009 0.143)**	**< .05**	**0.009 (0.003 0.014)**	**< .01**	**-0.004 (-0.007 -0.000)**	**< .05**
**Maternal Education [reference: beyond high school]**								
Less than high school	0.107 (-0.647 0.860)	> .05	0.252 (-0.334 0.837)	> .05	0.037 (-0.013 0.086)	> .05	0.016 (-0.013 0.044)	> .05
Completed high school	0.133 (-0.509 0.776)	> .05	-0.259 (-0.758 0.240)	> .05	-0.012 (-0.054 0.030)	> .05	-0.000 (-0.025 0.024)	> .05
EEG epoch analyzed	-0.004 (-0.029 0.021)	> .05	0.016 (-0.003 0.036)	> .05	0.000 (-0.002 0.002)	> .05	-0.001 (-0.002 0.000)	> .05
Females	**EEG Frequency Bands**							
Predictors	**Theta (µV²)**		**Alpha (µV²)**		**Beta (µV²)**		**Gamma (µV²)**	
**PAE [reference: non-drinking]**								
Quit-early drinking	0.029 (-0.228 0.286)	> .05	0.189 (-0.019 0.397)	> .05	0.009 (-0.008 0.026)	> .05	-0.001 (-0.009 0.008)	> .05
Continuous drinking	-0.127 (-0.755 0.501)	> .05	-0.126 (-0.634 0.328)	> .05	-0.010 (-0.052 0.032)	> .05	-0.014 (-0.034 0.006)	> .05
**PTE [reference: non-smoking]**								
Quit-early smoking	0.598 (-0.052 1.248)	> .05	0.414 (-0.112 0.939)	> .05	-0.022 (-0.066 0.006)	> .05	**-0.021 (-0.042 -0.000)**	**< .05**
Continuous smoking	0.122 (-0.459 0.702)	> .05	-0.004 (-0.474 0.466)	> .05	-0.010 (-0.029 0.049)	> .05	**0.024 (0.005 0.043)**	**< .05**
**Age at EEG assessment**	**-0.177 (-0.236 -0.117)**	**< .001**	0.016 (-0.032 0.064)	> .05	**0.004 (0.000 0.008)**	**< .01**	**-0.002 (-0.004 -0.001)**	**< .05**
**Maternal Education [reference: beyond high school]**								
Less than high school	-0.301	> .05	0.014	> .05	0.010	> .05	**0.029**	**< .01**

© 2024 Pini N et al. *JAMA Network Open.*

	(-0.894 0.292)		(-0.446 0.493)		(-0.030 -0.050)		**(0.010 0.048)**	
Completed high school	0.076 (-0.305 0.457)	> .05	-0.097 (-0.405 0.211)	> .05	-0.001 (-0.027 0.024)	> .05	0.009 (-0.003 0.021)	> .05
EEG epoch analyzed	-0.012 (-0.033 0.008)	> .05	-0.004 (-0.021 0.013)	> .05	**-0.002 (-0.003 -0.000)**	**< .05**	-0.000 (-0.001 0.000)	> .05

© 2024 Pini N et al. *JAMA Network Open.*

Sensitivity Analyses

Two types of sensitivity analyses were conducted to assess the stability of the associations between PAE and PTE clusters and EEG power. As illustrated in the manuscript, EEG was collected more than once in a subset of the participants. Specifically, N=5 participants contributed EEG recordings at both 4- and 5-years-of-age; N=25 participants contributed EEG recordings at both 4- and 7-years-of-age; N=34 participants contributed EEG recordings at both 5- and 7-years-of-age; N=3 participants contributed EEG recordings at both 5- and 9-years-of-age; N=15 participants contributed EEG recordings at both 7- and 9-years-of-age; N=1 participants contributed EEG recordings at both 9- and 11-years-of-age; and N=28 participants contributed EEG recordings at both 4-, 5, and 7-years-of-age.

Sensitivity Analysis #1

For this first sensitivity analysis, for participants contributing more than a single EEG recording, only the least recent observation was retained.

eTable 4 reports the associations of PAE and PTE clusters on EEG Power in EO.

eTable 5 reports the associations of PAE and PTE clusters on EEG Power in EC.

Sensitivity Analysis #2

An alternative approach sensitivity to analysis #1 was performed by utilizing generalized estimating equation (GEE) model clustering at the individual level. Within this framework, participants contributing more than a single observation were retained in the model by taking into account the notion that a subset of the complete set of observations is correlated (participants whose EEG activity was collected at multiple timepoints – please see a description of the distribution of repeated measures above).

eTable 6 reports the associations of PAE and PTE clusters on EEG Power in EO.

eTable 7 reports the associations of PAE and PTE clusters on EEG Power in EC.

© 2024 Pini N et al. *JAMA Network Open.*

eTable 6. Estimates (marginal means (CI) and *p*-values) of the associations of PAE and PTE and EEG power (EO blocks only) in sensitivity analysis #1

	EEG Frequency Bands							
Predictors	**Theta (μV²)**		**Alpha (μV²)**		**Beta (μV²)**		**Gamma (μV²)**	
**PAE [reference: non-drinking]**								
Quit-early drinking	0.046 (-0.089 0.182)	> .05	**0.109 (0.002 0.215)**	**< .05**	0.011 (-0.006 0.028)	> .05	0.004 (-0.007 0.016)	> .05
Continuous drinking	0.110 (-0.191 0.411)	> .05	**0.141 (0.002 0.280)**	**< .05**	0.023 (-0.014 0.060)	>.05	-0.010 (-0.035 0.016)	> .05
**PTE [reference: non-smoking]**								
Quit-early smoking	-0.094 (-0.397 0.210)	> .05	-0.086 (-0.324 0.153)	> .05	-0.020 (-0.057 0.017)	> .05	-0.021 (-0.047 0.005)	> .05
Continuous smoking	-0.047 (-0.303 0.209)	> .05	-0.113 (-0.314 0.088)	> .05	**-0.030 (-0.060 -0.001)**	**< .05**	**-0.020 (-0.038 -0.002)**	**< .05**
**Age at EEG assessment**	**-0.139 (-0.168 -0.110)**	**< .001**	**-0.032 (-0.055 -0.008)**	**< .001**	-0.000 (-0.004 0.003)	> .05	**-0.005 (-0.007 -0.002)**	**< .001**
**Sex assignment at birth [reference: male]**	**-0.461 (-0.593 -0.329)**	**< .001**	**-0.232 (-0.336 -0.128)**	**< .01**	**-0.057 (-0.073 -0.041)**	**< .001**	**-0.027 (-0.039 -0.016)**	**< .001**
**Maternal Education [reference: beyond high school]**								
Less than high school	0.047 (-0.225 0.319)	> .05	0.111 (-0.104 0.325)	> .05	**0.045 (0.012 0.079)**	**< .001**	**0.048 (0.025 0.072)**	**< .001**
Completed high school	0.009 (-0.206 0.223)	> .05	-0.030 (-0.199 0.139)	> .05	-0.004 (-0.031 0.022)	> .05	0.003 (-0.015 0.022)	> .05
EEG epoch analyzed	**-0.012 (-0.023 -0.001)**	**< .05**	0.004 (-0.005 0.012)	> .05	**-0.002 (-0.003 -0.001)**	**< .001**	**-0.002 (-0.003 -0.001)**	**< .001**

© 2024 Pini N et al. *JAMA Network Open.*

eTable 7. Estimates (marginal means (CI) and *p*-values) of the associations of PAE and PTE and EEG power (EC blocks only) in sensitivity analysis #1

	EEG Frequency Bands							
Predictors	Theta (μV^2)		Alpha (μV^2)		Beta (μV^2)		Gamma (μV^2)	
**PAE [reference: non-drinking]**								
Quit-early drinking	-0.088 (-0.342 0.167)	> .05	**0.215 (0.012 0.417)**	**< .01**	0.009 (-0.009 0.026)	> .05	-0.001 (-0.010 0.008)	> .05
Continuous drinking	-0.082 (-0.652 0.488)	> .05	**0.442 (0.050 0.834)**	**< .05**	**0.040 (0.001 0.078)**	**< .01**	-0.013 (-0.033 0.008)	> .05
**PTE [reference: non-smoking]**								
Quit-early smoking	0.190 (-0.385 0.765)	> .05	0.119 (-0.338 0.576)	> .05	-0.005 (-0.044 0.034)	> .05	-0.006 (-0.026 0.015)	> .05
Continuous smoking	-0.348 (-0.833 0.137)	> .05	-0.333 (-0.718 0.053)	> .05	-0.012 (-0.045 0.021)	> .05	0.013 (-0.004 0.030)	> .05
**Age at EEG assessment**	**-0.241 (-0.296 -0.186)**	**< .001**	0.037 (-0.007 0.081)	> .05	**0.005 (0.002 0.009)**	**< .01**	**-0.003 (-0.005 -0.001)**	**< .001**
**Sex assignment at birth [reference: male]**	**-0.738 (-0.986 -0.490)**	**< .001**	**-0.604 (-0.801 -0.407)**	**< .001**	**-0.055 (-0.071 -0.038)**	**< .001**	**-0.022 (-0.031 -0.013)**	**< .001**
**Maternal Education [reference: beyond high school]**								
Less than high school	0.053 (-0.467 0.573)	> .05	0.059 (-0.355 0.473)	> .05	0.027 (-0.008 0.062)	> .05	**0.027 (0.009 0.046)**	**< .01**
Completed high school	0.085 (-0.316 0.486)	> .05	-0.182 (-0.501 0.137)	> .05	-0.007 (-0.034 0.020)	> .05	0.006 (-0.009 0.020)	> .05
EEG epoch analyzed	-0.007 (-0.026 0.012)	> .05	0.009 (-0.006 0.024)	> .05	-0.001 (-0.002 0.001)	> .05	-0.001 (-0.001 0.000)	> .05

© 2024 Pini N et al. *JAMA Network Open.*

eTable 8. Estimates (marginal means (CI) and *p*-values) of the associations of PAE and PTE and EEG power (EO blocks only) in sensitivity analysis #2

	**EEG Frequency Bands**							
Predictors	**Theta (μV²)**		**Alpha (μV²)**		**Beta (μV²)**		**Gamma (μV²)**	
**PAE [reference: non-drinking]**								
Quit-early drinking	0.009<br>(-0.123 0.141)	> .05	**0.116<br>(0.01 0.217)**	**< .05**	0.012<br>(-0.003 0.026)	> .05	0.003<br>(-0.007 0.012)	> .05
Continuous drinking	0.131<br>(-0.195 0.456)	> .05	**0.211<br>(0.001 0.421)**	**< .05**	0.018<br>(-0.027 0.0)	>.05	-0.011<br>(-0.033 0.011)	> .05
**PTE [reference: non-smoking]**								
Quit-early smoking	-0.121<br>(-0.381 0.139)	> .05	-0.079<br>(-0.297 0.140)	> .05	-0.014<br>(-0.046 0.019)	> .05	**-0.021<br>(-0.041 -0.001)**	**< .05**
Continuous smoking	-0.064<br>(-0.282 0.154)	> .05	-0.118<br>(-0.278 0.041)	> .05	**-0.031<br>(-0.058 -0.004)**	**< .01**	**-0.020<br>(-0.039 -0.000)**	**< .05**
**Age at EEG assessment**	**-0.138<br>(-0.165 -0.112)**	**< .001**	**-0.030<br>(-0.049 -0.010)**	**< .01**	0.000<br>(-0.003 0.004)	> .05	**-0.004<br>(-0.006 -0.002)**	**< .001**
**Sex assignment at birth [reference: male]**	**-0.476<br>(-0.606 -0.347)**	**< .001**	**-0.237<br>(-0.338 -0.137)**	**< .001**	**-0.054<br>(-0.068 -0.040)**	**< .001**	**-0.031<br>(-0.040 -0.021)**	**< .001**
**Maternal Education [reference: beyond high school]**								
Less than high school	-0.016<br>(-0.296 0.263)	> .05	0.105<br>(-0.093 0.304)	> .05	0.041<br>(-0.000 0.082)	> .05	**0.044<br>(0.002 0.086)**	**< .01**
Completed high school	-0.018<br>(-0.221 0.186)	> .05	-0.007<br>(-0.161 0.148)	> .05	-0.002<br>(-0.022 0.018)	> .05	0.004<br>(-0.010 0.016)	> .05
EEG epoch analyzed	**-0.016<br>(-0.029 -0.003)**	**< .05**	0.003<br>(-0.003 0.010)	> .05	**-0.002<br>(-0.003 -0.000)**	**< .01**	**-0.002<br>(-0.003 -0.001)**	**< .001**

© 2024 Pini N et al. *JAMA Network Open.*

eTable 9. Estimates (marginal means (CI) and *p*-values) of the associations of PAE and PTE and EEG power (EC blocks only) in sensitivity analysis #2

	EEG Frequency Bands							
Predictors	**Theta (μV²)**		**Alpha (μV²)**		**Beta (μV²)**		**Gamma (μV²)**	
**PAE [reference: non-drinking]**								
Quit-early drinking	-0.170 (-0.419 0.078)	> .05	**0.203 (0.013 0.393)**	**< .05**	0.008 (-0.007 0.023)	> .05	-0.001 (-0.010 0.005)	> .05
Continuous drinking	0.173 (-0.502 0.848)	> .05	**0.385 (0.011 0.781)**	**< .05**	0.038 (-0.019 0.010)	> .05	-0.008 (-0.026 0.008)	> .05
**PTE [reference: non-smoking]**								
Quit-early smoking	0.144 (-0.536 0.824)	> .05	0.022 (-0.407 0.450)	> .05	-0.003 (-0.032 0.026)	> .05	-0.006 (-0.025 0.013)	> .05
Continuous smoking	-0.313 (-0.640 0.014)	> .05	**-0.309 (-0.579 -0.040)**	**< .05**	-0.014 (-0.044 0.015)	> .05	0.012 (-0.012 0.036)	> .05
**Age at EEG assessment**	**-0.236 (-0.284 -0.188)**	**< .001**	**0.043 (0.004 0.083)**	**< .05**	**0.006 (0.002 0.010)**	**< .001**	**-0.003 (-0.005 -0.000)**	**< .001**
**Sex assignment at birth [reference: male]**	**-0.779 (-1.020 -0.533)**	**< .001**	**-0.567 (-0.760 -0.374)**	**< .001**	**-0.050 (-0.070 -0.034)**	**< .001**	**-0.024 (-0.032 -0.016)**	**< .001**
**Maternal Education [reference: beyond high school]**								
Less than high school	-0.036 (-0.525 0.453)	> .05	0.124 (-0.196 0.444)	> .05	0.024 (-0.008 0.056)	> .05	0.022 (-0.004 0.048)	> .05
Completed high school	0.096 (-0.282 0.474)	> .05	-0.128 (-0.436 0.180)	> .05	-0.003 (-0.024 0.018)	> .05	0.006 (-0.006 0.018)	> .05
EEG epoch analyzed	-0.007 (-0.022 0.009)	> .05	0.011 (-0.000 0.021)	> .05	-0.000 (-0.001 0.000)	> .05	**-0.000 (-0.001 -0.000)**	**< .05**

© 2024 Pini N et al. *JAMA Network Open.*

eFigure 1. Participant Recruitment Flowchart

eFigure 1. CONSORT diagram

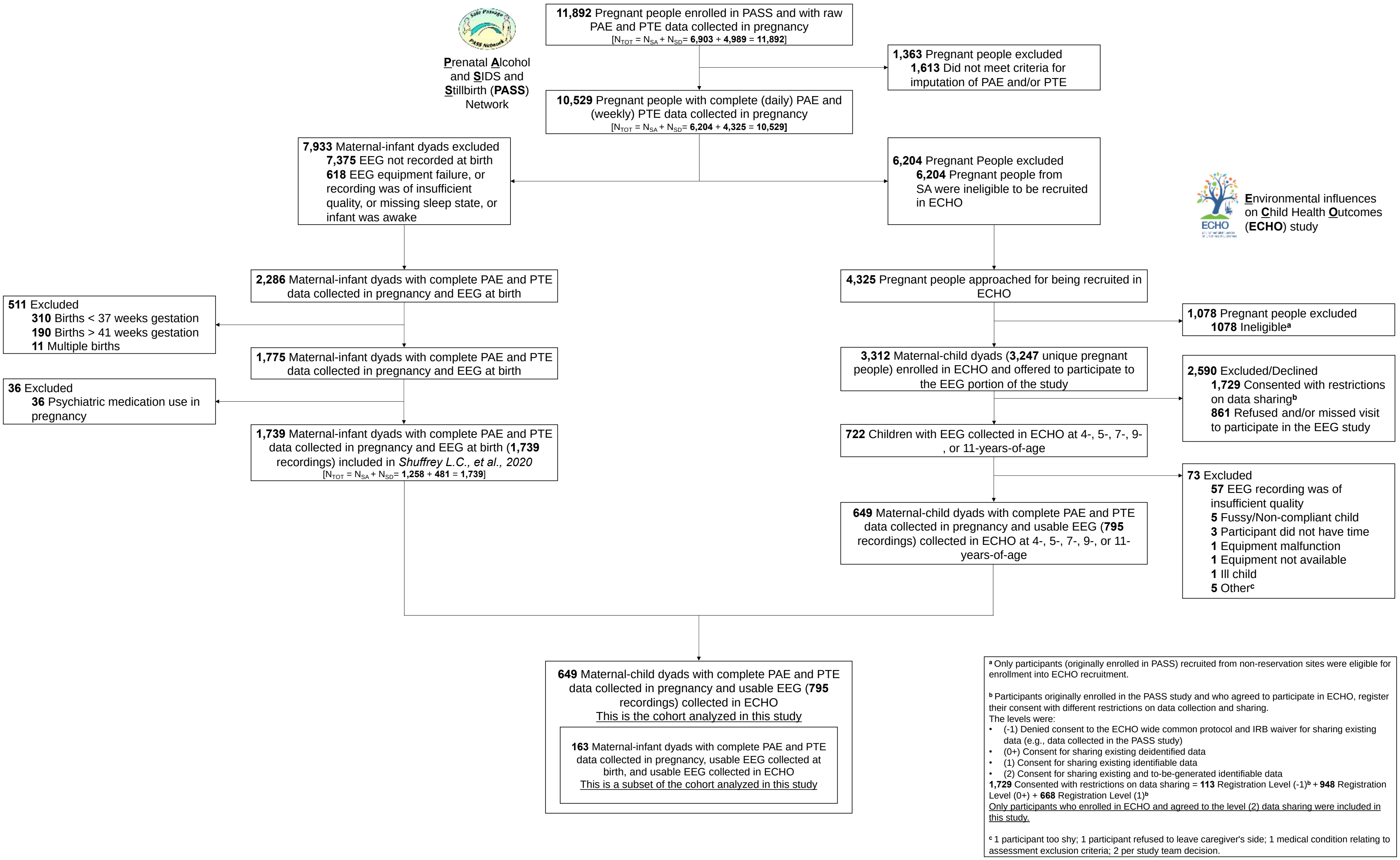

© 2024 Pini N et al. *JAMA Network Open.*

eReferences

1. Debnath R, Buzzell GA, Morales S, Bowers ME, Leach SC, Fox NA. The Maryland analysis of developmental EEG (MADE) pipeline. *Psychophysiology*. Jun 2020;57(6):e13580. doi:10.1111/psyp.13580
2. Delorme A, Makeig S. EEGLAB: an open source toolbox for analysis of single-trial EEG dynamics including independent component analysis. *J Neurosci Methods*. Mar 15 2004;134(1):9-21. doi:10.1016/j.jneumeth.2003.10.009
3. Nolan H, Whelan R, Reilly RB. FASTER: Fully Automated Statistical Thresholding for EEG artifact Rejection. *J Neurosci Methods*. Sep 30 2010;192(1):152-62. doi:10.1016/j.jneumeth.2010.07.015
4. Leach SC, Morales S, Bowers ME, et al. Adjusting ADJUST: Optimizing the ADJUST algorithm for pediatric data using geodesic nets. *Psychophysiology*. Aug 2020;57(8):e13566. doi:10.1111/psyp.13566
5. Mognon A, Jovicich J, Bruzzone L, Buiatti M. ADJUST: An automatic EEG artifact detector based on the joint use of spatial and temporal features. *Psychophysiology*. Feb 2011;48(2):229-40. doi:10.1111/j.1469-8986.2010.01061.x
6. Perrin F, Pernier J, Bertrand O, Echallier JF. Spherical splines for scalp potential and current density mapping. *Electroencephalogr Clin Neurophysiol*. Feb 1989;72(2):184-7. doi:10.1016/0013-4694(89)90180-6
7. Osselton JW. Acquisition of EEG data by bipolar, unipolar and average reference methods: a theoretical comparison. *Electroencephalogr Clin Neurophysiol*. Nov 1965;19(5):527-8. doi:10.1016/0013-4694(65)90195-1
8. Ostlund B, Donoghue T, Anaya B, et al. Spectral parameterization for studying neurodevelopment: How and why. *Dev Cogn Neurosci*. Apr 2022;54:101073. doi:10.1016/j.dcn.2022.101073
9. Donoghue T, Haller M, Peterson EJ, et al. Parameterizing neural power spectra into periodic and aperiodic components. *Nat Neurosci*. Dec 2020;23(12):1655-1665. doi:10.1038/s41593-020-00744-x
10. McSweeney M, Morales S, Valadez EA, et al. Age-related trends in aperiodic EEG activity and alpha oscillations during early- to middle-childhood. *Neuroimage*. Apr 1 2023;269:119925. doi:10.1016/j.neuroimage.2023.119925
11. Isler JR, Pini N, Lucchini M, et al. Longitudinal characterization of EEG power spectra during eyes open and eyes closed conditions in children. *Psychophysiology*. Jan 2023;60(1):e14158. doi:10.1111/psyp.14158

© 2024 Pini N et al. *JAMA Network Open.*
