# Supplementary material for: In Utero Exposure to Alcohol and Tobacco and Electroencephalogram Power During Childhood
Source: JAMA Netw Open. 2024 Jan 5;7(1):e2350528. doi: 10.1001/jamanetworkopen.2023.50528 (PMC10770777; doi:10.1001/jamanetworkopen.2023.50528)
Supplement: Supplement 2. — Data Sharing Statement [file jamanetwopen-e2350528-s002.pdf]

Data Sharing Statement

Pini. In Utero Exposure to Alcohol and Tobacco and Electroencephalogram Power During Childhood. *JAMA Netw Open*. Published January 05, 2024.
doi:10.1001/jamanetworkopen.2023.50528

Data
Data available: Yes
Data types: Deidentified participant data
How to access data: Nicolò Pini had full access to all the data presented in this study and takes responsibility for the integrity of the data and the accuracy of the data analysis. The data that support the findings of this study are available from the corresponding author, Nicolò Pini, upon reasonable request to be reviewed by the study team. Beginning date of data availability: with publication. Corresponding Author: Nicolò Pini, Department of Psychiatry, Columbia University Irving Medical Center, 1051 Riverside Drive, Room 4911C, New York, New York 10032, Email address: np2600@cumc.columbia.edu and Telephone: +1 (646) 774-6239
When available: With publication

Supporting Documents
Document types: None

Additional Information
Who can access the data: Nicolò Pini had full access to all the data presented in this study and takes responsibility for the integrity of the data and the accuracy of the data analysis. The data that support the findings of this study are available from the corresponding author, Nicolò Pini, upon reasonable request to be reviewed by the study team. Beginning date of data availability: with publication. Corresponding Author: Nicolò Pini, Department of Psychiatry, Columbia University Irving Medical Center, 1051 Riverside Drive, Room 4911C, New York, New York 10032, Email address: np2600@cumc.columbia.edu and Telephone: +1 (646) 774-6239
Types of analyses: Nicolò Pini had full access to all the data presented in this study and takes responsibility for the integrity of the data and the accuracy of the data analysis. The data that support the findings of this study are available from the corresponding author, Nicolò Pini, upon reasonable request to be reviewed by the study team. Beginning date of data availability: with publication. Corresponding Author: Nicolò Pini, Department of Psychiatry, Columbia University Irving Medical Center, 1051 Riverside Drive, Room 4911C, New York, New York 10032, Email address: np2600@cumc.columbia.edu and Telephone: +1 (646) 774-6239
Mechanisms of data availability: Nicolò Pini had full access to all the data presented in this study and takes responsibility for the integrity of the data and the accuracy of the data analysis. The data that support the findings of this study are available from the corresponding author, Nicolò Pini, upon reasonable request to be reviewed by the study team. Beginning date of data availability: with publication. Corresponding Author: Nicolò Pini, Department of Psychiatry, Columbia University Irving Medical Center, 1051 Riverside Drive, Room 4911C, New York, New York 10032, Email address: np2600@cumc.columbia.edu and Telephone: +1 (646) 774-6239
Any additional restrictions: Nicolò Pini had full access to all the data presented in this study and takes responsibility for the integrity of the data and the accuracy of the data analysis. The data that support the findings of this study are available from the corresponding author, Nicolò Pini, upon reasonable request to be reviewed by the study team. Beginning date of data availability: with publication. Corresponding Author: Nicolò Pini, Department of Psychiatry, Columbia University Irving Medical Center, 1051 Riverside Drive, Room 4911C, New York, New York 10032, Email address: np2600@cumc.columbia.edu and Telephone: +1 (646) 774-6239
